# *MIR205HG*/*LEADR* Long Noncoding RNA Binds to Primed Proximal Regulatory Regions in Prostate Basal Cells Through a Triplex- and *Alu*-Mediated Mechanism

**DOI:** 10.3389/fcell.2022.909097

**Published:** 2022-06-17

**Authors:** Eugenia Bezzecchi, Giulia Pagani, Barbara Forte, Stefano Percio, Nadia Zaffaroni, Diletta Dolfini, Paolo Gandellini

**Affiliations:** ^1^ Department of Biosciences, University of Milan, Milan, Italy; ^2^ Center for Omics Sciences, IRCCS San Raffaele Scientific Institute, Milan, Italy; ^3^ Molecular Pharmacology Unit, Fondazione IRCSS Istituto Nazionale dei Tumori, Milan, Italy

**Keywords:** long noncoding RNA, triplex, *Alu*, ChIRP, sequencing

## Abstract

Aside serving as host gene for *miR-205*, *MIR205HG* transcribes for a chromatin-associated long noncoding RNA (lncRNA) able to restrain the differentiation of prostate basal cells, thus being reannotated as *LEADR* (Long Epithelial *Alu*-interacting Differentiation-related RNA). We previously showed the presence of *Alu* sequences in the promoters of genes modulated upon *MIR205HG*/*LEADR* manipulation. Notably, an *Alu* element also spans the first and second exons of *MIR205HG*/*LEADR*, suggesting its possible involvement in target selection/binding. Here, we performed ChIRP-seq to map *MIR205HG*/*LEADR* chromatin occupancy at genome-wide level in prostate basal cells. Our results confirmed preferential binding to regions proximal to gene transcription start site (TSS). Moreover, enrichment of triplex-forming sequences was found upstream of *MIR205HG*/*LEADR*-bound genes, peaking at −1,500/−500 bp from TSS. Triplexes formed with one or two putative DNA binding sites within *MIR205HG*/*LEADR* sequence, located just upstream of the *Alu* element. Notably, triplex-forming regions of bound genes were themselves enriched in *Alu* elements. These data suggest, from one side, that triplex formation may be the prevalent mechanism by which *MIR205HG*/*LEADR* selects and physically interacts with target DNA, from the other that direct or protein-mediated *Alu* (RNA)/*Alu* (DNA) interaction may represent a further functional requirement. We also found that triplex-forming regions were enriched in specific histone modifications, including H3K4me1 in the absence of H3K27ac, H3K4me3 and H3K27me3, indicating that in prostate basal cells *MIR205HG*/*LEADR* may preferentially bind to primed proximal regulatory elements. This may underscore the need for basal cells to keep *MIR205HG*/*LEADR* target genes repressed but, at the same time, responsive to differentiation cues.

## Introduction

Long noncoding RNAs (lncRNAs) are an interesting class of transcripts longer than 200 nt, which are devoid of protein-coding potential though exerting important regulatory functions in a variety of biological processes (Ulitsky and Bartel, 2013) and disease states (Rossi et al., 2020; Pandini et al., 2021; Tassinari and Gandellini, 2021; Cava et al., 2022). Differently from mRNAs, they can be expressed either in the nucleus or in the cytoplasm (or both), an aspect, that is, intimately linked with their mechanism of action. In fact, depending on their subcellular localization, their activities range from regulating the chromatin state and transcription in the nucleus to acting post-transcriptionally as sponges for microRNAs in the cytoplasm (Statello et al., 2021). This wide spectrum of regulation modalities is intrinsically linked to their RNA nature. Indeed, the interactome of RNA may include other nucleic acids, both DNA and RNA, to which RNA can bind through canonical Watson-Crick base pairing (as is the case of RNA/RNA duplexes or R-loops with the DNA) or Hoogsteen bonds, as in triple helices or triplexes. In addition, by folding into complex secondary and tertiary structures, RNA can provide scaffolds for interacting with proteins (Tassinari et al., 2021). Although examples for all the cited modes of action have been reported, most lncRNAs are still poorly characterized from the mechanistic point of view.

In this regard, *MIR205HG* is a lncRNA abundantly expressed in epithelia, the function of which has been acknowledged only recently. In physiological conditions, our group showed that, aside serving as host gene for *miR-205* (Gandellini et al., 2012), *MIR205HG* acts as nuclear lncRNA able to restrain the differentiation of prostate basal cells, a finding that led to its reannotation as *LEADR* (Long Epithelial *Alu*-interacting Differentiation-related RNA) (Profumo et al., 2019). An oncogenic role has been proposed for *MIR205HG* in several cancers characterized by the expansion of basal cells, such as cervical (Li et al., 2019), lung squamous cell (Liu et al., 2020), head and neck squamous cell carcinomas (Di Agostino et al., 2018). A tumor-suppressive function has been instead reported in esophageal adenocarcinoma, where *MIR205HG* is downregulated (Song et al., 2021; Dong et al., 2022). From the mechanistic side, most of the studies suggest that *MIR205HG* would act post-transcriptionally as sponge for various miRNAs, among which *miR-122-5p*, *miR-299-3p* (Guo et al., 2021) and *miR-590-3p*, or by hindering the translation of *HNRNPA0* mRNA (Dong et al., 2022). However, data from the lncAtlas (Mas-Ponte et al., 2017) (Supplementary Figure S1) and from our group (Profumo et al., 2019) indicate that the expression of *MIR205HG* is prevalently nuclear and chromatin-associated.

Here, to start elucidating *MIR205HG*/*LEADR* mechanism of functioning at the chromatin level, we performed Chromatin Isolation by RNA purification (ChIRP)-sequencing (Chu et al., 2011) of immortalized prostate epithelial cells, which express the lncRNA at high level. Our results show that *MIR205HG*/*LEADR* preferentially binds to primed proximal regulatory regions by forming DNA/RNA triplexes, through a mechanism that may imply the participation of *Alu* elements present in both the lncRNA and target genes.

## Materials and Methods

### Chromatin Isolation by RNA Purification-Sequencing

ChIRP was performed on RWPE-1 cells in triplicate as described in Profumo et al*.* (2019). RWPE-1 cells were chosen as model of normal prostate basal epithelial cells, as they express the typical basal cytokeratins (*KRT5* and *KRT14*) together with high levels of *MIR205HG*/*LEADR* (Profumo et al., 2019). A unique pool of ten 20-mer 3′-BiotinTEG-modified antisense probes covering the whole sequence of *MIR205HG*/*LEADR* transcript (1 probe/100 bp of RNA length) was used for the experiment, whereas a symmetrical set of probes against *lacZ* RNA was used as mock control (sequences are reported in Profumo et al., 2019). RNA was obtained from ChIRP-ed samples to check successful enrichment of *MIR205HG*/*LEADR* transcript upon precipitation with specific probes as compared to *lacZ* probes (Supplementary Figure S2A). DNA from the same samples was obtained for sequencing. High-throughput sequencing libraries from three independent ChIRP experiments were prepared and indexed using the ThruPLEX^®^ DNA-seq Kit (Rubicon Genomics), purified on AMPure XP beads, then checked for quality and size range on Agilent Bioanalyzer using the High Sensitivity DNA Assay kit. Samples were then pooled and sequenced on Illumina HiSeq2000 with single read length of 100 bp (SR100). Raw reads were aligned to the human genome Hg19 using Bowtie2. Raw data and detailed procedures have been made publicly available on GEO, with accession GSE201567.

### Peak Calling, Filtering, and Annotation

Peaks of each *MIR205HG*-ChIRP sample were called against *LacZ* signal using MACS2, with *p*-value cut-off of 1e-5. For each MACS2 peak, we filtered for regions sharing the same features in at least two of three independent experiments using the *findConsensusPeakRegions* function of the consensusSeekeR package v1.18.0 (Samb et al., 2015) in R environment (R version 4.0.3). We provided two inputs: peaks (files containing called peaks) and narrowPeaks (files containing called peak regions), both given by MACS2 as output. The package consensusSeekeR compares genomic positions and genomic ranges from multiple experiments to extract common regions. We took advantage of this tool and created consensus peaks extending the region of 500 bp on both sides of the position of the peak center. We shrank the region size, which is set by the *extendingSize* parameter, to fit the narrow peak regions of the peaks when all the regions were smaller than the consensus region through the *shrinkToFitPeakRegion* parameter. We finally obtained 5,064 consensus peaks, considered as the definitive peaks, characterized by an average length of 775 bp. Annotated consensus peaks are reported in Supplementary Table S1. The tracks of narrow peaks of single replicates and of consensus peaks are depicted in Supplementary Figure S2B. Enrichment of genomic features within consensus peaks was assessed using the *annotatePeak* function of ChipSeeker R package v1.26.2 giving −1,500; +500 from the transcription start site (TSS) as our custom definition of “proximal regions.” The TxDb was built from the Ensembl database, consequently the Ensembl annotation (EnsDb.Hsapiens.v75 v2.99.0) of transcripts was used. As a control, 1,000 lists of 5,000 random peaks of 775 bp of length (i.e., average length of *MIR205HG* peaks) were created with the bedtools toolset. We also run *annotatePeaks.pl* of HOMER software with default parameters (Human Genome as background) in order to compare results with the enrichments obtained by ChipSeeker.

### Enrichment Analyses

We employed LOLA v1.24.0 (Sheffield and Bock, 2016), a Bioconductor package, to perform the enrichment analysis for genomic region sets and regulatory elements. We used the *buildRestrictedUniverse* function to build a universe based on query sets and to test for differential enrichment of regions against a background. As a control, a list of 500 random peaks of 775 bp of length falling in proximal regulatory regions (−1,500; +500 from the TSS) was created with the bedtools toolset. The tested Region Databases included LOLA standard features, collection of *Alu* sequences downloaded from Table Browser (hg19) and ChIP-seq data for histone modification patterns in RWPE-1 cells (GSE63094).

### Triplex Prediction and Enrichment

We used Regulatory Genomics Toolbox (RGT) (http://www.regulatory-genomics.org/tdf/basic-introduction/) (Kuo et al., 2019; Sentürk et al., 2019), an open-source python library, in order to find the triplex-forming potential between RNA and DNA regions of 15–20 base pair length (Kuo et al., 2019). We run the genomic region test function of the Triplex Domain Finder (TDF) tool, by providing the coordinates of consensus peaks in bed format and *MIR205HG* sequence (ENST00000429156.1). We also compared our results with different controls such as *MEG3* peaks or a random list of 500 sequences spanning −1,500 to −500 bp from TSS. With the Sfold tool (https://sfold.wadsworth.org/cgi-bin/index.pl) (Ding and Lawrence, 2001; Ding and Lawrence, 2003; Zuker, 2003; Ding et al., 2004), we identified and then masked *MIR205HG* bases having less than 10% of probability of being single-stranded. The promoter test of TDF was used for the list of genes differentially expressed upon *MIR205HG* manipulation (GSE104003).

### Statistical Analyses

All bioinformatic analyses were performed in the R environment (R version 4.0.3). Statistical analysis was performed using Chi-square test to assess the difference between expected and observed frequencies and Jonckheere-Terpstra (JT) test to assess the monotone trend of different classes. When applying permutation analysis, empiric *p*-value was calculated as 1 divided for the number of times the observed percentage of a given feature in the set of random gene lists was statistically significantly different (based on multiple testing-corrected Chi-square *p*-value) from the percentage of the tested peak list. A threshold of 0.05 was considered statistically significant.

## Results

### *MIR205HG*/*LEADR* Binds to Proximal Regulatory Regions Through Triplex Formation

**FIGURE 1:**
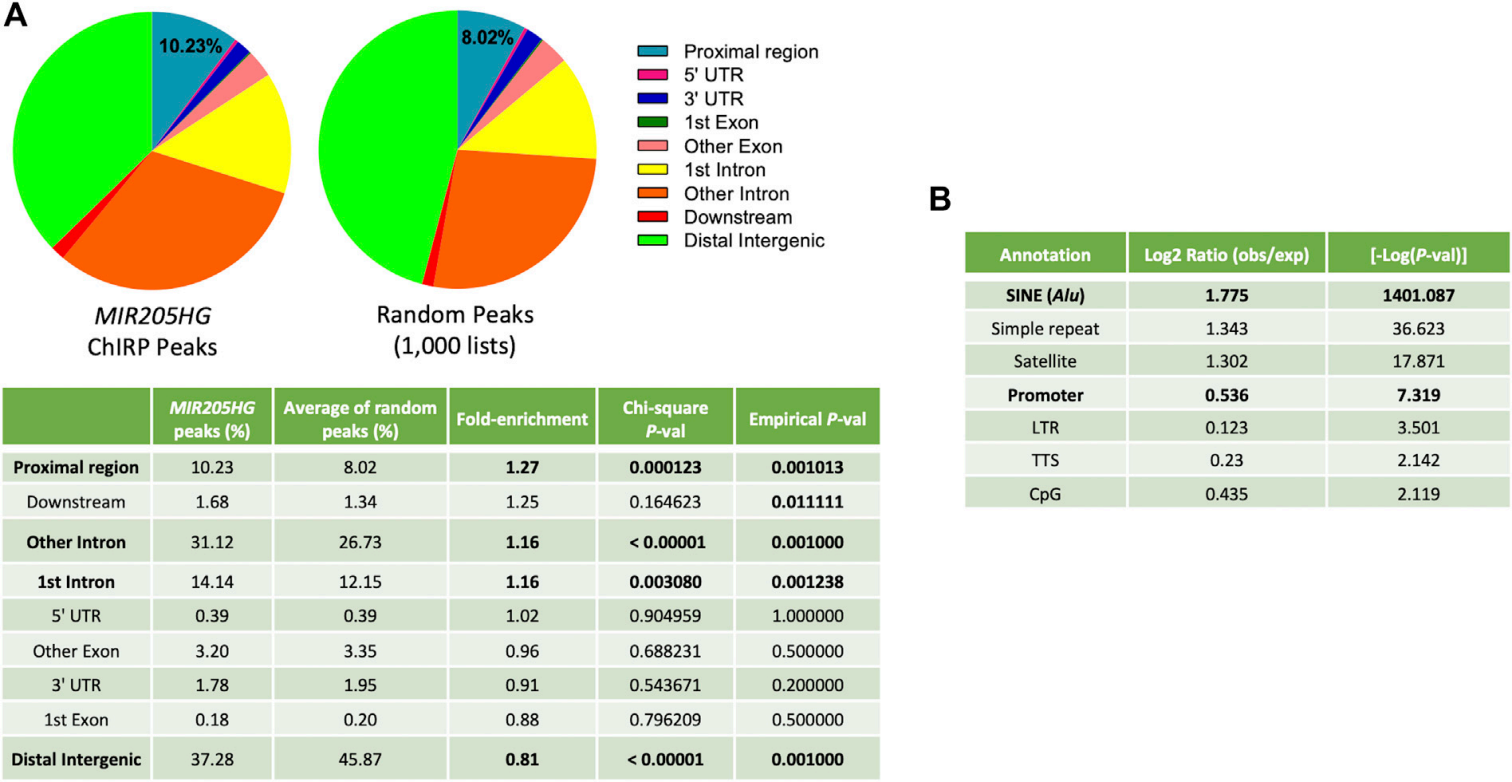
Genome distribution of *MIR205HG* ChIRP consensus peaks as compared to random regions. **(A)** Feature enrichment analysis performed using the *annotatePeak* function of *ChipSeeker* R package on 5,064 consensus peaks and on 1,000 lists of about 5,000 random peaks of equal nucleotide length. The pie plots were generated using GraphPad prism. Chi-square test *p*-values calculated against the average percentage of each feature in random gene lists and empirical *p*-values associated with permutation analyses are reported in the enclosed table. Proximal Region = −1,500 to +500 bp from gene TSS; Downstream = within 3,000 bp from gene termination. **(B)** Feature enrichment analysis performed using the *annotatePeaks.pl* function of HOMER software on 5,064 consensus peaks. The tables were generated using Microsoft Excel.

To map the chromatin occupancy of *MIR205HG*/*LEADR* at a genome-wide level in prostate basal cells, three independent *MIR205HG*-ChIRP experiments were performed on immortalized RWPE-1 cells, as described in Profumo et al. (2019) and subjected to high-throughput sequencing. Actual pull-down of *MIR205HG* (and not *GAPDH*) RNA with specific probes as compared to *lacZ* probes is reported for the three replicate experiments in Supplementary Figure S2A. Peaks in *MIR205HG*-ChIRP samples were called against the *lacZ*-ChIRP sample to correct for non-specific binding. Then consensus peaks shared by at least two out of three experiments were selected, resulting in 5,064 unique peaks (Supplementary Figure S2B, Supplementary Table S1). Such peaks were distributed all over the genome, however they overlapped more preferentially to regions proximal to gene TSS (−1,500 to +500 bp, hereafter defined as “proximal regions”) than to all other regions, when tested against 1,000 lists of 5,000 random regions of comparable length (775 bp) (∼1.3-fold enrichment: 10.23% in *MIR205HG* ChIRP peaks vs. 8.02% of averaged random regions, Chi-square test *p* = 0.000123; empirical *p*-value calculated upon permutation of random gene lists *p* = 0.0001) (Figure 1A). A similar analysis run with the HOMER tool confirmed significant (−log*P* = 7.3) enrichment of promoters (defined by the tool as −1,000 bp to +100 bp, so included in our “proximal regions”) and unearthed even higher enrichment (−log*P* = 1401) of SINE/*Alu* sequences, as compared to the background (Figure 1B). Overall, such observation is in trend with the enrichment of *Alu* sequences found in promoters of genes differentially expressed upon *MIR205HG* manipulation (Profumo et al., 2019). Notably, *MIR205HG* peaks were also significantly enriched in intronic regions, suggesting that the lncRNA may bind outside of proximal regulatory elements (Figure 1A). In this work, we decided to focus our attention on the specific features of *MIR205HG* binding to proximal regions.

**FIGURE 2:**
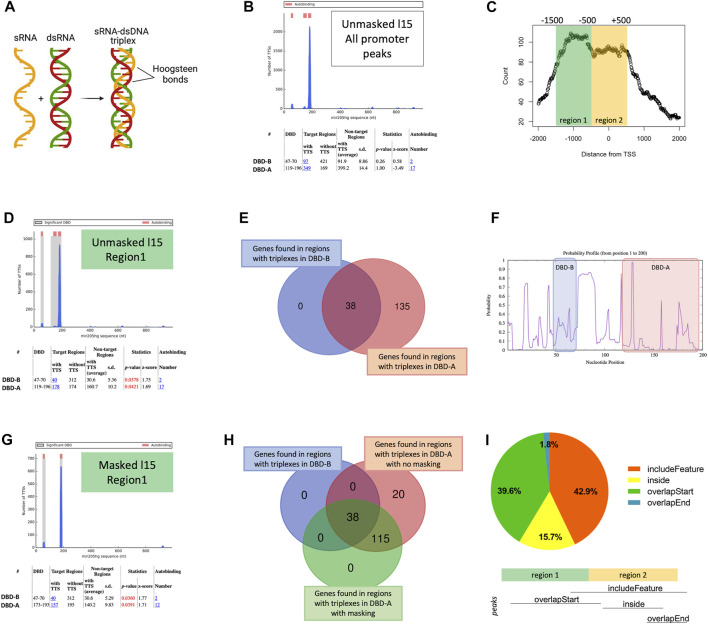
Enrichment of triplexes in *MIR205HG* proximal region peaks. **(A)** Representation of RNA/DNA triplex formation (created with BioRender.com). **(B)** TDF output for the unmasked analysis with minimum triplex length equal to 15 performed on 518 *MIR205HG* proximal region peaks. **(C)** Bimodal distribution of *MIR205HG* proximal region peaks around the TSS, generated using the *binOverFeature* function of the *ChIPpeakAnno* R package (v3.28.1). **(D)** TDF output of the unmasked analysis with minimum triplex length equal to 15 performed on “region 1”-cutpeaks. **(E)** Intersection between genes forming a triplex with 119–196 DBD-A (173) and genes forming a triplex with 47–70 DBD-B (38) of *MIR205HG* transcript. **(F)** Profile showing the probability of *MIR205HG* bases (form base position 1 to 200) of being single stranded. The DBD-B and DBD-A are highlighted. The image was generated by Sfold tool. **(G)** TDF output of the masked analysis with minimum triplex length equal to 15 performed on “region 1”-cutpeaks. **(H)** Intersection between genes forming a triplex with DBD-A (173) of *MIR205HG* with no masking, genes forming a triplex with DBD-B (38) and with 173–196 DBD-A with masking. **(I)** Degree of overlap between whole peaks and region 2 (−500/+500 bp from TSS) calculated using the *findOverlappingPeaks* function of the *ChIPpeakAnno* R package (v3.28.1). Results show that 42.9% of peaks included region 2 completely, though starting before (i.e., in region 1). This is indicated in the figure as “includeFeature”. 39.6% of peaks fell mainly in region 1, only overlapping with the start of region 2 (“overlapstart”); 15.7% fell completely within region 2 (“inside”); 1.8% of peaks overlapped with the end of region 2 (“overlapend”). Pie plot was generated using GraphPad prism. Venn diagrams were generated with the web accessible tool available at https://bioinformatics.psb.ugent.be/webtools/Venn/.

One of the emerging mechanisms by which lncRNAs seem to interact with chromatin is DNA/RNA triplexes (Figure 2A). In these structures, the single-stranded RNA of the lncRNA accommodates into the double helix of the DNA by forming Hoogsteen bonds [Reviewed in (Li et al., 2016)]. Therefore, we wondered whether *MIR205HG* could bind to the target DNA through the formation of triplexes. The analysis run with TDF tool (using 15 nt as the minimum triplex size) on the 518 peaks in proximal regions (corresponding to 448 genes), identified 349 regions able to form triplexes (the so-called DNA binding sites, DBS) with *MIR205HG* (Figure 2B), however their frequency was not significantly different from that of non-target regions.

The analysis of ChIRP-seq read counts with respect to TSS however showed a bimodal distribution of proximal region peaks (Figure 2C), with higher density from −1,500 to −500 bp (region 1) and lower between −500 and +500 bp (region 2). Therefore, we generated “region 1” and “region 2” -cutpeaks as a result of the intersection between proximal region peaks and the abovementioned regions. Interestingly, TDF found significant enrichment of triplex-forming sequences in the first region (Figure 2D), and lack of enrichment in the latter (with even fewer triplexes than expected) (Supplementary Figure S3A). Specifically, DBS were found in 178 of 352 “region 1”-cutpeaks, corresponding to 173 genes. These DBS formed triplexes with 119–196 nt region of *MIR205HG*, hereafter referred to as putative DNA binding domain A (DBD-A). Notably, 40 out of 178 (=22.5%) “region 1”-cutpeaks forming a triplex with DBD-A were predicted to form an additional triplex with 47–70 nt region of *MIR205HG* (hereafter referred to as putative DBD-B), meaning that all DBS forming a triplex with DBD-B also form a triplex with the more downstream DBD-A, corresponding to 38 out of 173 genes (Figure 2E). Notably, DBD-A was confirmed to be significantly enriched in “region 1”-cutpeaks (49 DBS in 352 peaks) when the analysis was run using 20 nt as minimum triplex length, whereas DBD-B was lost (Supplementary Figure S3B).

Importantly, no significant enrichment of triplex-forming regions with *MIR205HG* was found in ChOP peaks of *MEG3*, an unrelated lncRNA known to form triplexes. Moreover, no triplex-forming regions with *MEG3* were found in *MIR205HG* peaks spanning −1,500 to −500 bp from TSS. The other way around, triplex-forming regions with *MIR205HG* were not statistically enriched in a random list of 500 sequences spanning −1,500 to −500 bp from TSS.

It is known that lncRNAs may be highly structured and that some bases may not be available for triplex formation due to their involvement in RNA secondary structures (Matveishina et al., 2020). Therefore, we masked *MIR205HG* bases having a <10% likelihood of being single-stranded and thus being unavailable to form triplexes, and re-run TDF analysis. Notably, bases ranging from 47 to 70 of *MIR205HG* (DBD-B) all appeared to exceed the cut-off of accessibility, whereas DBD-A resulted to be highly accessible starting from base 174 (Figure 2F). TDF run using masked *MIR205HG* sequence again revealed enrichment of DBS in “region 1”-cutpeaks (Figure 2G), with 157 triplexes forming with DBD-A (all included in the 178 triplexes found in the “unmasked” analysis) and 40 (of 40 of the unmasked analysis) with DBD-B of *MIR205HG* (Figure 2H). It is to note that, according to this analysis, DBD-A appeared to be shorter, spanning bases 173–193 of *MIR205HG* sequence.

Overall these data suggest that *MIR205HG* peaks from −1,500 to −500 of TSS may account for a triplex-forming mechanism, whereas −500/+500 peaks may be alternatively region 1 peak tails or accounting for a different mechanism of binding. To explore this, we verified the degree of overlap between whole peaks and region 2. We found that 42.9% of peaks covered both regions (i.e., started in region 1 but included region 2 completely) whereas 39.6% and 17.5% (15.7% + 1.8%) of peaks fell specifically in region 1 and in region 2, respectively (Figure 2I). The existence of region-specific peaks may underscore the possibility that *MIR205HG* may bind to the DNA with different mechanisms, being however triplex formation the most prevalent modality (total peaks covering region 1 = 42.9% + 39.6% = 82.5%).

### *MIR205HG*/*LEADR* Triplex-Forming Peaks are Enriched in *Alu* Sequences and Histone Modifications of Primed Regulatory Elements

**FIGURE 3:**
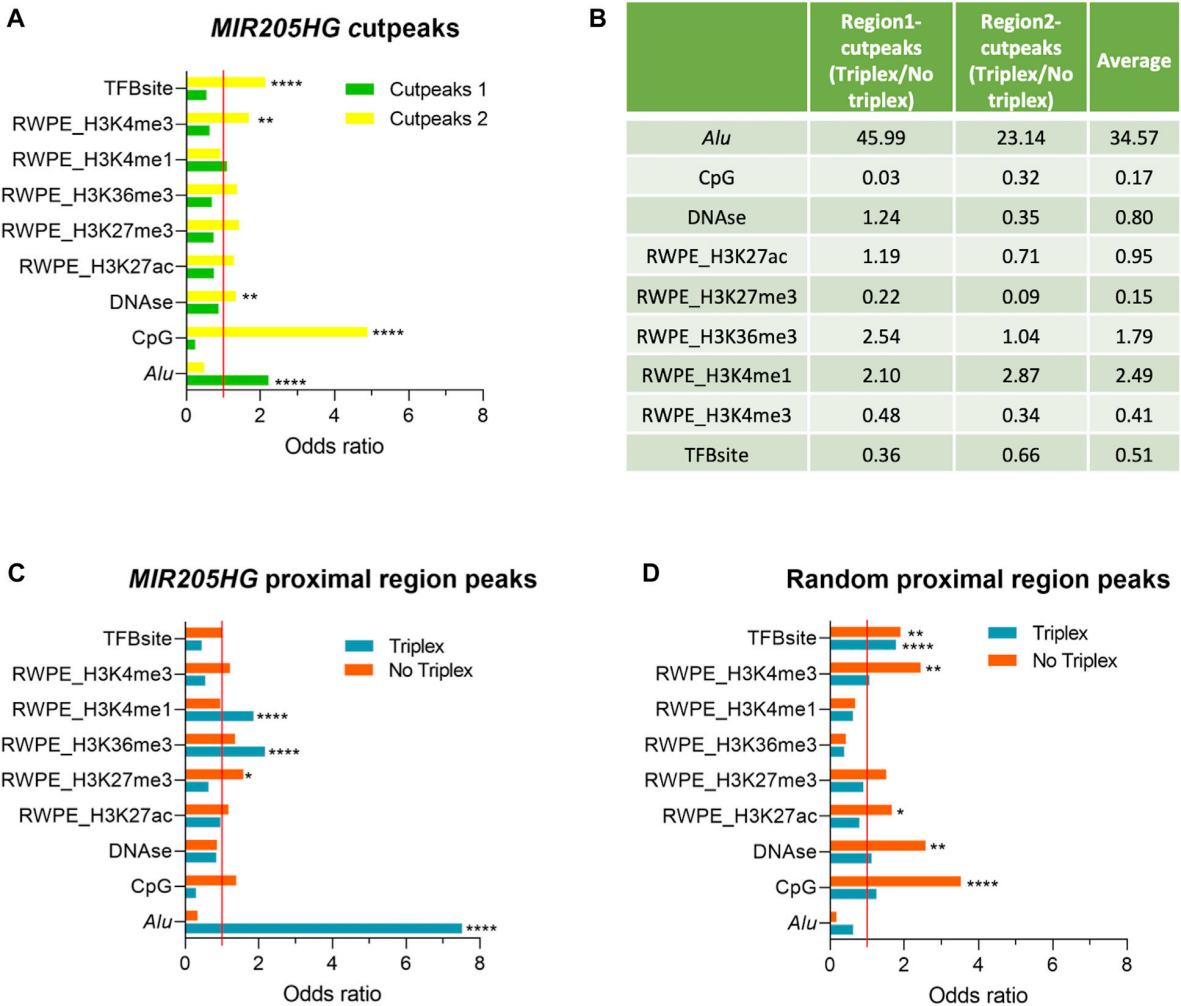
Enrichment of *Alu* and specific histone modifications in *MIR205HG* proximal region peaks. **(A)** The bar plot shows the odds ratios for the “region 1”- and “region 2”-cutpeaks for each LOLA library. Line set to 1 represents no enrichment as compared to the background. **(B)** For each LOLA library, the ratio of the odds ratios in triplex-containing and in triplex-less peaks among “region 1” and “region 2”-cutpeaks, together with their average, are reported to show the relative enrichment in presence and absence of the triplex. **(C)** The bar plot shows the odds ratios for *MIR205HG* proximal region peaks divided into triplex-forming and triplex-less peaks for each LOLA library. **(D)** The bar plot shows the odds ratios for random proximal region peaks divided into triplex-forming and triplex-less peaks for each LOLA library. The computed *p*-Value is reported (*p < 0.05, **p < 0.01, ***p < 0.001, ****p < 0.0001). Bar plots and the table were generated using GraphPad Prism and Microsoft Excel, respectively.

We then used LOLA to highlight features that could be specific to either bound region. We found exclusive enrichment of *Alu* sequences in “region 1”-cutpeaks and of transcription factor binding sites (TFBsite), DNAse, CpG islands, H3K4me3 in “region 2”-cutpeaks (Figure 3A). All other tested features resulted to be not significantly enriched overall in cutpeaks as compared to the background (Figure 3A). To exclude that feature enrichments could be region-specific and not associated with triplex formation, we run a comparative analysis between triplex-containing and triplex-less peaks in each region. In both cases, triplex-containing peaks were enriched in *Alu* and H3K4me1 and devoid of CpG, H3K27me3, and H3K4me3 (Figure 3B). In region 1, they were also enriched in H3K36me3 and devoid of TFBS. These results seem to confirm that some feature enrichments are specific of *MIR205HG* modality of binding (triplex yes vs*.* no), rather than being solely region-specific. Potentially, this may also underscore a dual mode of *MIR205HG* binding, the first and more prevalent involving triplex formation in proximity of *Alu* sequences and H3K4me1 modification, more frequently occurring in region 1; the latter, possibly mediated by the interaction with TFs and accompanied by H3K4me3 and H3K27me3, more frequently occurring in region 2.

We focused on the triplex-mediated mechanism and to test it from a more general perspective, we went back to the analysis of all proximal region peaks. To increase specificity, we run TDF with 0.1 masking on whole peaks (not “cut” for regions). As previously observed in the unmasked analysis, we did not find any significant enrichment of triplexes overall, however we could detect 306 triplexes forming with site DBD-A (in 283 genes) and 97 with site DBD-B (in 93 genes, all included in those forming triplex with DBD-A) (Supplementary Figure S3C). Overall, 59% of peaks had at least a triplex, which accounts for 63% of genes.

The LOLA analysis run on all proximal region peaks based on the presence/absence of the triplex, regardless of peak position with respect to TSS, confirmed enrichment of *Alu*, H3K4me1 and H3K36me3 in triplex-containing peaks, and H3K27me3 in triplex-less peaks (Figure 3C).

As a further validation, we run TDF to find triplex-forming sequences in a list of 500 random regions in the range of −1,500/+500 from TSS and then analyzed with LOLA. Notably, no enrichment of *Alu*, H3K4me1 or H3K36me3 was found in this case (Figure 3D), suggesting that 1) triplexes predicted in *MIR205HG* peaks may be true and that 2) features associated with them are specifically linked to *MIR205HG* mode of binding. For example, striking enrichment of *Alu* was found exclusively in *MIR205HG* peaks with triplex and not in triplex-less *MIR205HG* peaks, nor in unrelated proximal regions regardless of potential predicted triplexes.

**FIGURE 4:**
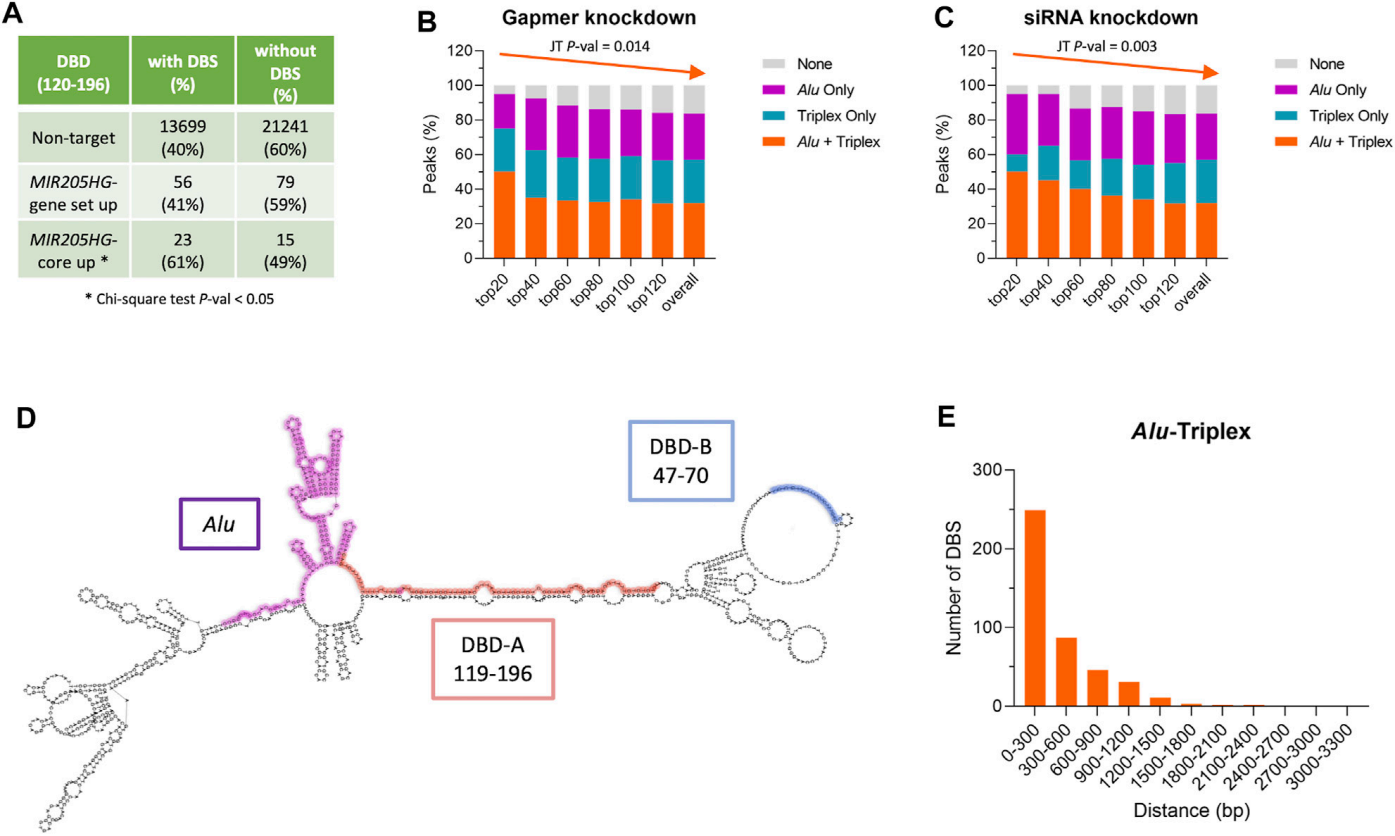
The presence of *Alu* and triplex-forming regions is a feature of both *MIR205HG* and its target genes. **(A)** Table showing the number and percentage of genes predicted to form or not a triplex with *MIR205HG* in lists of non-target genes (“Non-target”), genes commonly up-modulated after *MIR205HG* knockdown using either a siRNA or a gapmer (“*MIR205HG*-gene set up”), and genes commonly upmodulated after *MIR205HG* knockdown and coherently downmodulated after *MIR205HG* overexpression (“*MIR205HG*-core up”). Gene lists are from Profumo et al. (2019). **(B)** Bar plot showing the percentage of genes having a DBS (i.e., a triplex) and/or *Alu* element among genes differentially expressed after *MIR205HG* knockdown using a gapmer antisense oligonucleotide. Significance of the monotone trend of triplex + *Alu* percentage in regulated genes ranked for fold-change (top20 genes have the highest fold-change) was assessed by Jonckheere-Terpstra (JT) test. **(C)** Bar plot showing the percentage of genes having a DBS (i.e., a triplex) and/or *Alu* elements among genes differentially expressed after *MIR205HG* knockdown using a siRNA. Significance of the monotone trend of triplex + *Alu* percentage in regulated genes ranked for fold-change (top20 genes have the highest fold-change) was assessed by Jonckheere-Terpstra (JT) test. **(D)** *MIR205HG* predicted secondary structure (as from RNAfold); DBD-A, DBD-B and *Alu* elements are highlighted in the structure. **(E)** Distance between the triplex (DBS) and the *Alu* element in *MIR205HG* proximal region peaks forming a triplex. Bar plots and the table were generated using GraphPad Prism and Microsoft Excel, respectively.

The results are reminiscent of the enrichment of *Alu* sequences that we found in proximal regions of genes deregulated upon *MIR205HG* manipulation, as from microarray analysis (Profumo et al., 2019; Percio et al., 2020). TDF analysis run on such gene lists revealed significant enrichment of triplex-forming sequences in *bona fide MIR205HG* target genes (i.e., “*MIR205HG*-core up”) as compared to non-target genes (Figure 4A), which formed triplexes with DBD element of the lncRNA. Moreover, the simultaneous presence of DBS and *Alu* element in gene proximal regions was prominent in most markedly modulated genes and tended to decrease with fold-change (Figures 4B,C).

Analysis of *MIR205HG* secondary structure (as from RNAfold web service, http://rna.tbi.univie.ac.at/cgi-bin/RNAWebSuite/RNAfold.cgi) (Gruber et al., 2008; Lorenz et al., 2011) revealed that the putative DBDs, which are located just upstream of the *Alu* element, are poorly structured (Figure 4D), thus making them prone to triplex formation. In contrast, the *Alu* element is highly structured, making the protein-mediated interaction with *Alu* sequence on target genes more plausible than the direct DNA/RNA pairing. Most *MIR205HG* target genes as from ChIRP-seq analysis revealed to have triplex/DBS at a distance of less than 300 nt from their *Alu* element (Figure 4E), which would allow interaction with DBD and *Alu* on *MIR205HG* without any need of bending of either the DNA or the RNA. This is exemplified in Supplementary Figure S4, where some examples of genes having *MIR205HG* peak in their proximal regions are reported, together with indication of their triplex-forming region (DBS) and *Alu* element.

Aside *Alu*, features enriched exclusively in *MIR205HG* triplex-forming regions (not in other regions, even not in triplex-forming regions of random proximal regions) were H3K4me1 and H3K36me3 (Figure 3C, Supplementary Figure S4). Other features previously found to be enriched in triplex-less *MIR205HG* peaks (CpG, H3K4me3, H3K27me3, Figure 3B) showed a tendency to be enriched in triplex-less regions also in random proximal regions (Figure 3D), thus not allowing us to draw specific conclusions on whether and how *MIR205HG* may work in a triplex-independent modality.

## Discussion

Understanding of lncRNA function passes through the identification of elements that allow RNA to interact with other molecules (Graf and Kretz, 2020; Ohyama et al., 2020). In some cases, such interaction is simply mediated by complementarity between nucleic acids, as in the miRNA sponging mechanism. In most circumstances, however, more complex secondary and tertiary structures need to form to allow interaction with double-stranded DNA or proteins (Tassinari et al., 2021). In this regard, the primary sequence of lncRNAs is only poorly informative of the function of a given lncRNA, and curiously also less conserved than higher-order structures (Johnsson et al., 2014). It is however conceivable that, just alike proteins, lncRNAs may act through discrete, modular functional elements (Przanowska et al., 2022). Recognition of such structural motifs is together the main obstacle and the key to define the exact mechanism of action of lncRNAs.

Our previous work demonstrated, for the first time, the pivotal role of the chromatin-associated lncRNA *MIR205HG*/*LEADR* in controlling the basal phenotype of prostate epithelial cells (Profumo et al., 2019). Understanding the mechanism of action of *MIR205HG*/*LEADR* will allow to dissect the processes governing differentiation of epithelial cells, which often appear deregulated in cancer (Ferrari and Gandellini, 2020), and eventually lay the foundations for the development of novel therapeutic approaches for tumors where *MIR205HG*/*LEADR* expression is deregulated (Di Agostino et al., 2018; Li et al., 2019; Liu et al., 2020; Song et al., 2021; Dong et al., 2022). For a discussion on the potential (yet to be disclosed) role of *MIR205HG* in prostate cancer refer to Profumo et al. (2019).

Here, by analyzing the genome-wide chromatin occupancy of *MIR205HG*/*LEADR* in prostate basal cells, we provide initial clues into *MIR205HG*/*LEADR* functional modules. Specifically, we recognized two regions potentially essential for interaction with chromatin: 1) a highly structured *Alu* (RNA) element that potentially binds to *Alu* (DNA) motifs on target gene proximal regions, likely through protein intermediates, and 2) a poorly structured DBD responsible to form triplexes with regions located upstream of target genes; such domain should allow the physical direct interaction with the DNA double helix and simultaneously provide specificity for target genes over the plethora of *Alu* elements in the human genome. While we had previously showed that deletion of *Alu* element from *MIR205HG* sequence abrogated its capability to regulate gene expression, at least in part by impairing binding to target gene proximal regions, the functionality and real contribution of the predicted DBDs to DNA binding through triplex formation remain to be validated through *ad hoc* experiments. These may include selective deletion of either DBDs followed by analysis of *MIR205HG* chromatin occupancy, as well as direct biochemical assays to assess formation of triplex with target genes (as in Mondal et al., 2015; Sentürk et al., 2019).

Accumulating evidence shows that the formation of triplexes, especially with target gene regulatory regions, is a mechanism shared by different nuclear lncRNAs, such as *MEG3* (Mondal et al., 2015)—used as control in our experiments—*HOTAIR* (Kalwa et al., 2016) and *KHPS1* (Blank-Giwojna et al., 2019). In this regard, Sentürk et al. (2019) have recently provided evidence of the existence and physiological relevance of DNA/RNA triplexes *in vivo*. In their work, the authors also showed that triplexes can form at active chromatin domains and *in trans* with distant genomic loci.

The possible role of *Alu* elements as functional domains of lncRNAs has been proposed in 2014 as the so-called RIDL hypothesis (where RIDL stands for Repeat Insertion Domain of LncRNAs), mainly based on the correlative evidence of enrichment of *Alu* in lncRNA exons (Johnson and Guigó, 2014; Kim et al., 2016). For some lncRNAs, the essential role of *Alu* domains in function has been proven by deletion experiments, as in the case of *ANRIL* (Holdt et al., 2013) and *MIR205HG*/*LEADR* (Profumo et al., 2019). From the mechanistic point of view, most of the literature focuses on the “cytoplasmic activities” of *Alu* RNA domains. Imperfect base-pairing between *Alu* elements in the 3′-UTR of mRNAs and *Alu* elements in cytoplasmic lncRNAs was shown to transactivate *STAU1*-mediated mRNA decay (Gong and Maquat, 2011). *Alu*-directed sense to antisense interaction was demonstrated to be the mechanism by which SINEUPs, a new functional class of natural antisense lncRNAs (Schein et al., 2016), select mRNAs with overlapping 5′-UTR to ultimately increase their translation, a process, that is, mediated by a distinct effector domain (Spinoza et al., 2021). Sequences enriched in *Alu* repeats have been also reported to drive nuclear localization of long RNAs in human cells (Lubelsky and Ulitsky, 2018), even if this is not the case of *MIR205HG* (Profumo et al., 2019). Regarding the “nuclear” activity of *Alu* elements, *Alu*/*Alu* direct pairing was proposed between *APTR* lncRNA and the promoter of *CDKN1A*/p21 (Negishi et al., 2014), as well as between the splicing-regulatory lncRNA *5S-OT* and its targets (Hu et al., 2016). In contrast, *Alu* elements in the human lincRNA-p21 need to adopt a conserved secondary structure to regulate RNA function and localization (Chillón and Pyle, 2016). Also the *Alu* element of *ANRIL* was shown to form a stem-loop structure. Whether structured *Alu* domains are the scaffolds for the protein-mediated interaction with other *Alu* elements or more in general are the platforms for effector proteins is an aspect that still needs extensive investigation.

The possible link between triplex formation and the presence of the *Alu* in either the lncRNA or target genes is also a poorly developed issue. In this regard, Sentürk et al. (2019) showed that a large fraction of triplex-forming RNAs is enriched with repeat elements, thus suggesting that repeat-derived sequences may represent functional domains that target regulatory RNAs to distant genomic regions. In addition, triplex-forming DNA sequences have a propensity to harbor significantly more SINE and LTR elements than control DNA, with *Alu* and ERVL subclasses being predominant. This finding supports the notion that repetitive DNA sequences might serve an important function in tethering regulatory RNAs to specific genomic regions, as is for *MIR205HG*/*LEADR*. Partially in contrast with these and our results are the findings by Bai et al. (2021), who reported *Alu* enrichment in RNA:DNA hybrids (R-loops), but depletion in DNA:DNA and RNA:DNA triplexes.

Our analysis showed that triplex-containing *MIR205HG*/*LEADR* peaks were enriched in H3K4me1 in the absence of H3K27ac, H3K4me3 and H3K27me3, a feature that has been historically associated with primed enhancers, i.e., enhancers characterized by accessible chromatin conformation despite being transcriptionally repressed (Calo and Wysocka, 2013). This evidence is reminiscent of what has been reported for super-lncRNAs, a class of tissue-restricted lncRNAs that target and contribute to the local chromatin organization of the super-enhancers (i.e., regions in the genome containing multiple enhancers which drive transcription of genes involved in cell identity). Such lncRNAs harbor a single triplex-forming repeat domain, which forms an RNA:DNA triplex with multiple anchor DNA sites originating from transposable elements within the super-enhancers (Soibam, 2017). Therefore, preferential binding to enhancer-like regions through a triplex- and *Alu*-based mechanism seems to be a shared feature between super-lncRNAs and *MIR205HG*. Notably, *Alu* elements are found in most enhancers, which may suggest them as proto-enhancers in primates (Feschotte, 2008). In this regard, we should acknowledge that the mentioned histone modification pattern can be found in both distal and proximal enhancers, as well as in promoters (Bae and Lesch, 2020). As an alternative to the classical positional definition, promoters and enhancers may be considered as a single class of transcriptional elements distinguished by different levels of transcription, which are then associated with different ratios between H3K4me1 and other histone modifications. Whether *MIR205HG*-bound elements work as proximal enhancers or promoters of the neighboring genes should be assessed for each individual gene using the appropriate assays. From a global perspective, the “primed” histone modification pattern found to be associated with *MIR205HG* at proximal regions may underscore the need of basal cells to keep *MIR205HG*/*LEADR* target genes repressed but, at the same time, responsive to differentiation cues. Curiously, another triplex-forming lncRNA, *MEG3*, was found to preferentially interact with promoter-distal sites enriched in H3K4me1 and H3K27me3, which are instead characteristic of poised enhancers (Calo and Wysocka, 2013).

Further investigation will be required to understand whether *MIR205HG*/*LEADR* is itself responsible for the recruitment of histone modifying complexes at bound sites, thus directly regulating the chromatin state of target genes, and through which structural domain. In this regard, other triplex-forming lncRNAs, such as *MEG3*, *HOTAIR* and *PARTICLE*, were shown to recruit PRC2 complexes to promoters of target genes to drive epigenetic silencing (O'Leary et al., 2015). The *Alu*-containing lncRNA ANRIL was itself shown to regulate target gene expression through histone methylation complexes. In all of the mentioned cases, the lncRNA structural motifs responsible to recruit and transport necessary regulators to promoter/enhancers of target genes have been either yet not identified or shown to be distinct from the triplex-forming domain. It is not even unlikely that *Alu* elements may themselves participate in this process.

Overall, here we provided initial clues into *MIR205HG*/*LEADR* possible mechanism of action at the chromatin level, further corroborating the emerging role of *Alu* elements as functional RNA domains of lncRNAs and key regulatory DNA elements in proximal regions. We also confirmed triplex formation as a prominent mechanism of DNA binding for chromatin-interacting lncRNAs. Further experimental validation is warranted to assess the real contribution of the suggested functional elements and modality of DNA binding, as well as the mechanism by which *MIR205HG* is then able to directly modulate the expression of target genes. If successful, such experiments may inform on the role that *MIR205HG*/*LEADR* may have in the differentiation of basal cells from various tissue contexts (e.g., breast, skin) and stimulate interest regarding the contribution of aberrant differentiation programs to epithelial carcinogenesis.

## Data Availability

The datasets presented in this study can be found in online repositories. The names of the repository/repositories and accession number(s) can be found below: https://www.ncbi.nlm.nih.gov/geo/query/acc.cgi?acc=GSE201567.
