# Supplementary material for: MIR205HG/LEADR Long Noncoding RNA Binds to Primed Proximal Regulatory Regions in Prostate Basal Cells Through a Triplex- and Alu-Mediated Mechanism
Source: Front Cell Dev Biol. 2022 Jun 17;10:909097. doi: 10.3389/fcell.2022.909097 (PMC9247157; doi:10.3389/fcell.2022.909097)
Supplement: Supplementary file 1 [file DataSheet1.pdf]

Supplementary Material

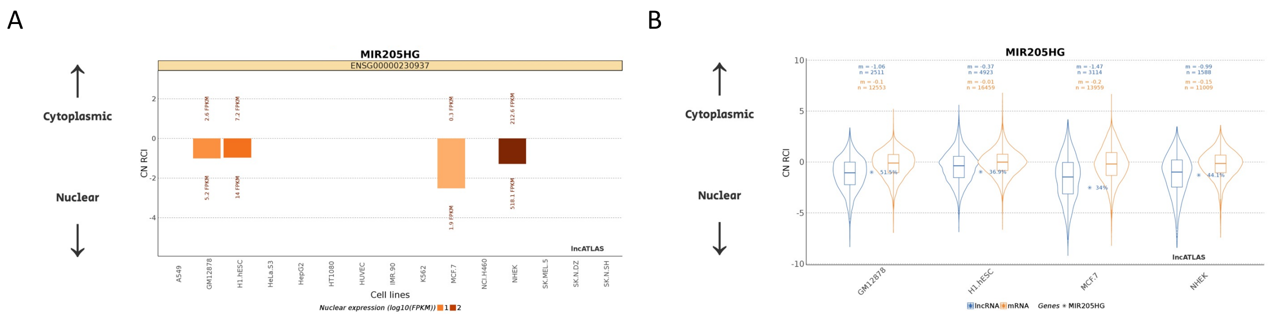

Supplementary Figure S1: *MIR205HG* is prevalently nuclear.

(A) Nuclear-cytoplasmic localization of *MIR205HG* as from lncATLAS. This localization is expressed in units of relative concentration index (RCI), which is a comparison of the concentration of a gene, per unit mass of RNA, between the two cellular compartments in different cell lines. Expression values are also reported.

(B) Nuclear-cytoplasmic localization of *MIR205HG* within the distribution of all genes expressed as RCI as from lncATLAS. *n* indicates the total number of genes in each group and *m* the median RCI value per group. The group percentile corresponding to each gene is also displayed next to the gene point.

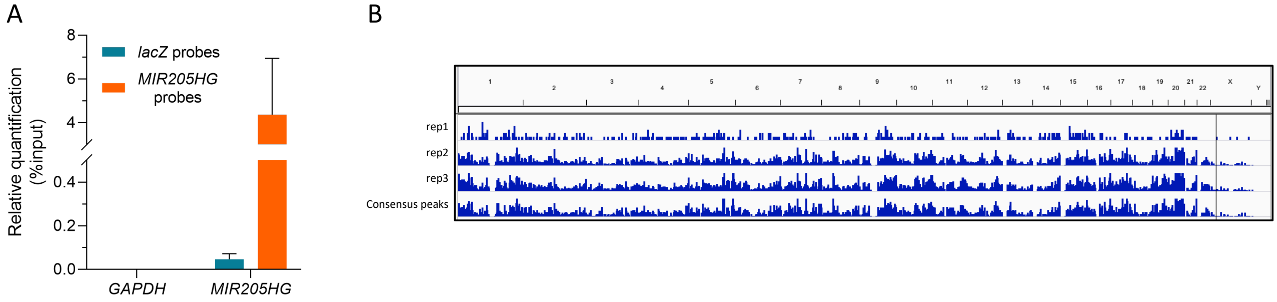

Supplementary Figure S2: ChIRP successfully precipitates *MIR205HG* RNA and bound DNA.

(A) Pull-down of *MIR205HG* (and not *GAPDH*) RNA with specific probes as compared to *lacZ* probes for the three replicate experiments subjected to sequencing (relative quantification of *GAPDH* and *MIR205HG* against the input in *lacZ* e *MIR205HG* probes). The graph was created with GraphPad Prism.

(B) Integrative Genomics Viewer (IGV) tracks reporting the distribution of *MIR205HG* ChIRP peaks across chromosomes, as called against *lacZ*. Signal of peaks from single replicates (rep1, rep2, and rep3) and of consensus peaks is reported.

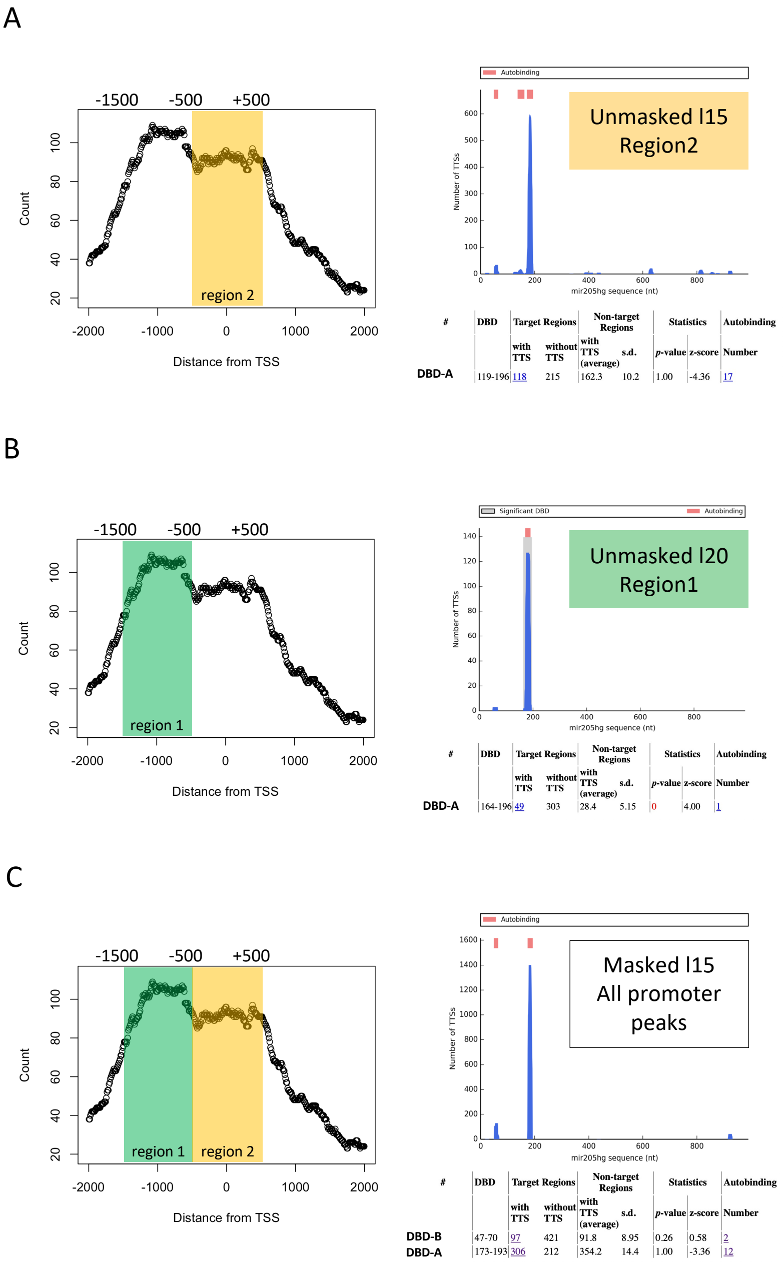

Supplementary Figure S3: Enrichment of triplexes in *MIR205HG* proximal region peaks.

(A) Highlight of region 2 (-500/+500 bp from TSS) of the bimodal distribution of proximal region peaks around the TSS (*left*). TDF output of the unmasked analysis with minimum triplex length equal to 15 performed on “region 2”-cutpeaks (*right*).

(B) Highlight of region 1 (-1,500/-500 bp from TSS) of the bimodal distribution of proximal region peaks around the TSS (*left*). TDF output of the unmasked analysis with minimum triplex length equal to 20 performed on “region 1”-cutpeaks (*right*).

(C) Highlight of both region 1 and region 2 of the bimodal distribution of proximal region peaks around the TSS (*left*). TDF output of the masked analysis with minimum triplex length equal to 15 performed on 518 promoter peaks (*right*).

A

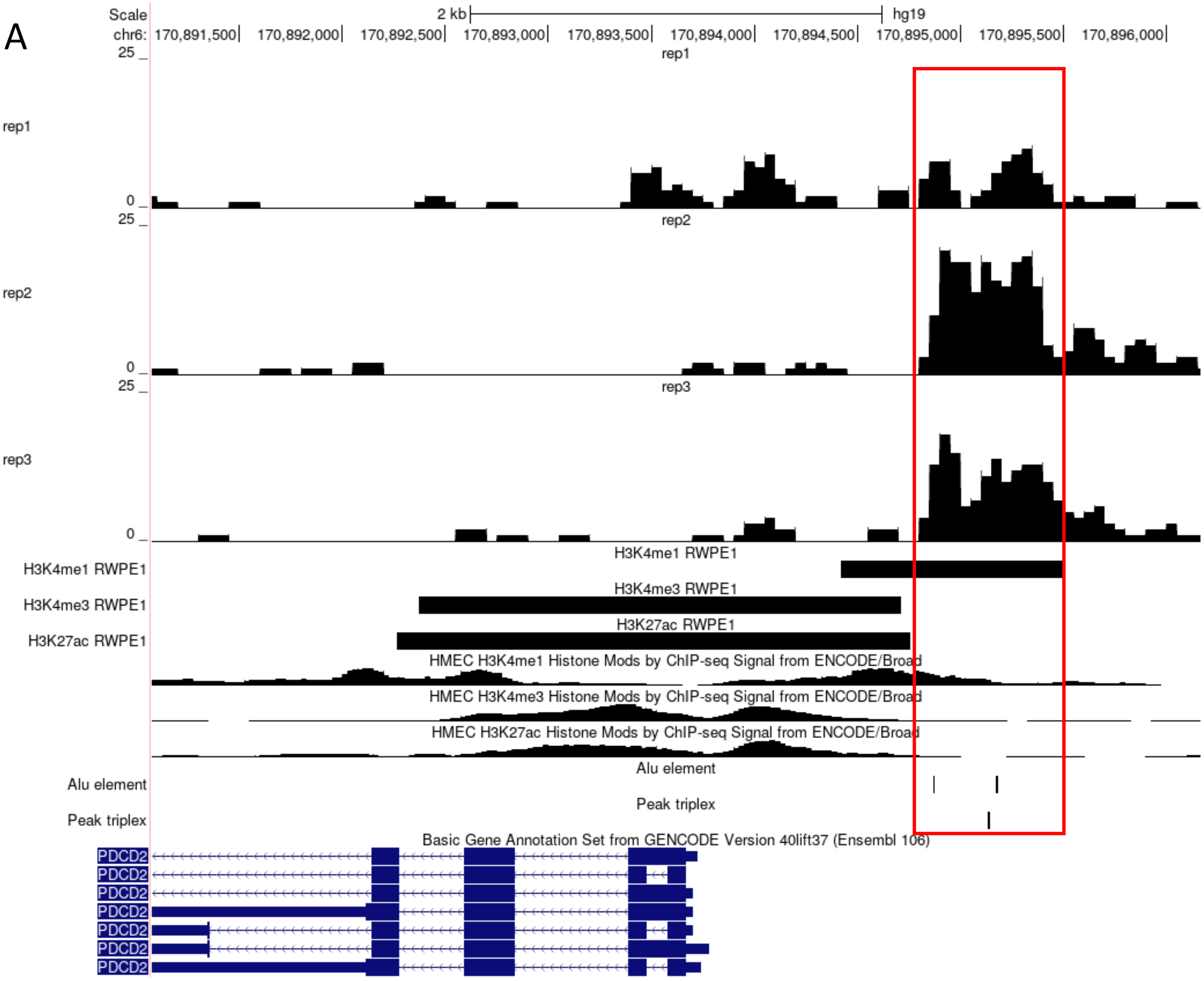
Scale
2 kb
hg19
chr6: 170,891,500| 170,892,000| 170,892,500| 170,893,000| 170,893,500| 170,894,000| 170,894,500| 170,895,000| 170,895,500| 170,896,000|
25 _
rep1
rep1
0 _
25 _
rep2
rep2
0 _
25 _
rep3
rep3
0 _
H3K4me1 RWPE1
H3K4me1 RWPE1
H3K4me3 RWPE1
H3K4me3 RWPE1
H3K27ac RWPE1
H3K27ac RWPE1
HMEC H3K4me1 Histone Mods by ChIP-seq Signal from ENCODE/Broad
HMEC H3K4me3 Histone Mods by ChIP-seq Signal from ENCODE/Broad
HMEC H3K27ac Histone Mods by ChIP-seq Signal from ENCODE/Broad
Alu element
Alu element
Peak triplex
Peak triplex
Basic Gene Annotation Set from GENCODE Version 40lift37 (Ensembl 106)
PDCD2
PDCD2
PDCD2
PDCD2
PDCD2
PDCD2
PDCD2

B

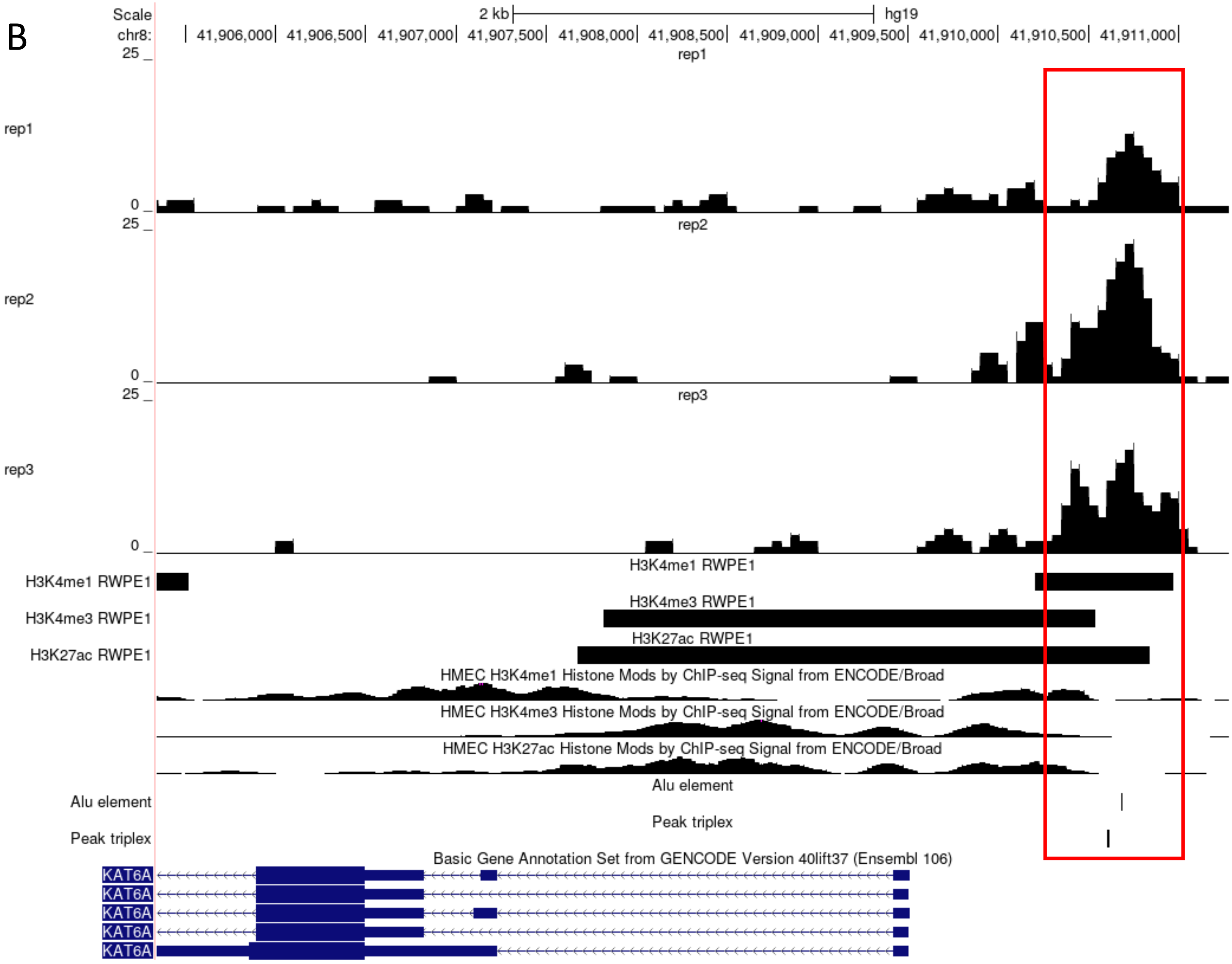

Scale
2 kb
hg19
chr8:
41,906,000
41,906,500
41,907,000
41,907,500
41,908,000
41,908,500
41,909,000
41,909,500
41,910,000
41,910,500
41,911,000
rep1
rep2
rep3
25
0
H3K4me1 RWPE1
H3K4me3 RWPE1
H3K27ac RWPE1
HMEC H3K4me1 Histone Mods by ChIP-seq Signal from ENCODE/Broad
HMEC H3K4me3 Histone Mods by ChIP-seq Signal from ENCODE/Broad
HMEC H3K27ac Histone Mods by ChIP-seq Signal from ENCODE/Broad
Alu element
Peak triplex
Basic Gene Annotation Set from GENCODE Version 40lift37 (Ensembl 106)
KAT6A

C

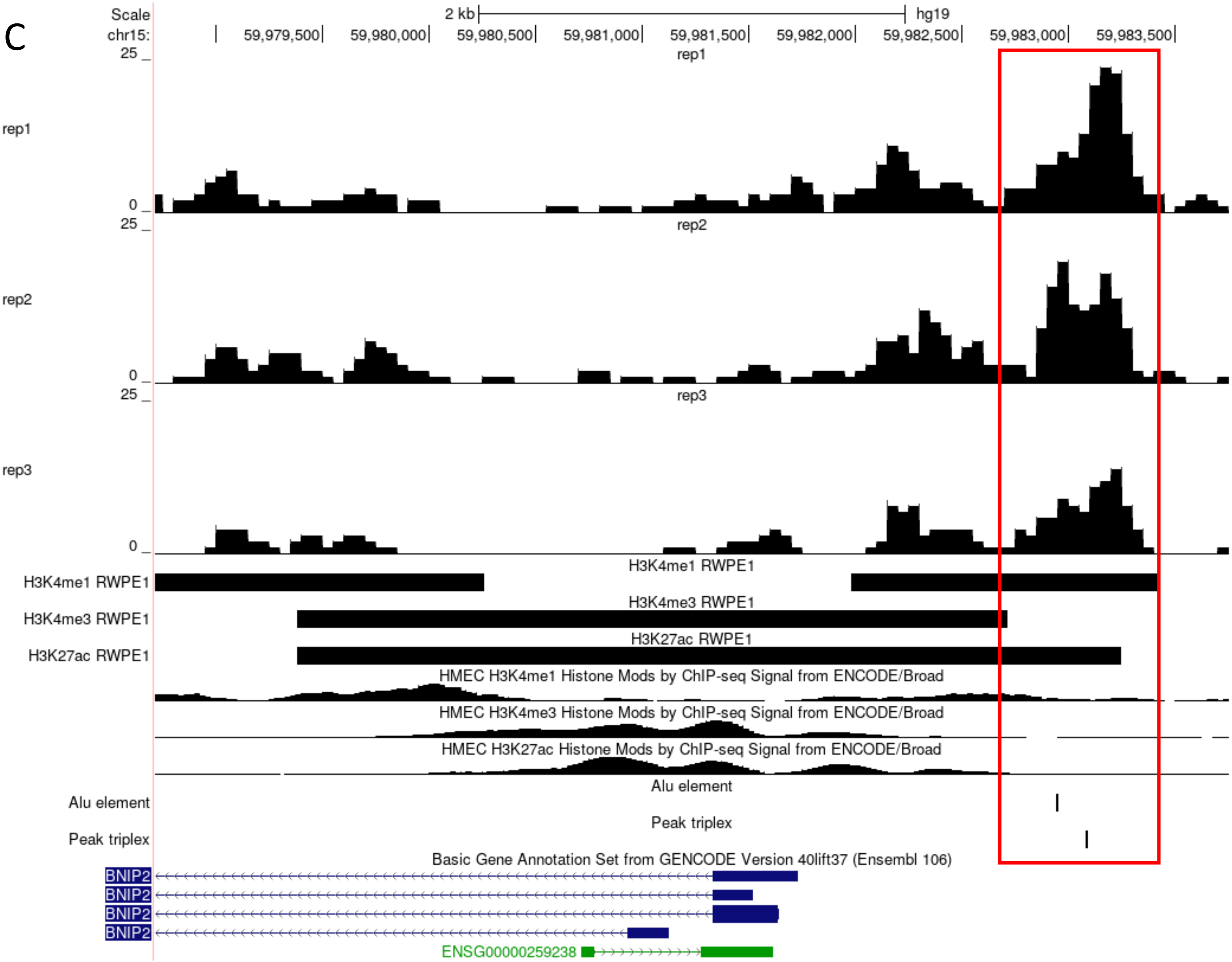
Scale
chr15:
2 kb
hg19
59,979,500
59,980,000
59,980,500
59,981,000
59,981,500
59,982,000
59,982,500
59,983,000
59,983,500
25
0
rep1
rep2
rep3
H3K4me1 RWPE1
H3K4me3 RWPE1
H3K27ac RWPE1
HMEC H3K4me1 Histone Mods by ChIP-seq Signal from ENCODE/Broad
HMEC H3K4me3 Histone Mods by ChIP-seq Signal from ENCODE/Broad
HMEC H3K27ac Histone Mods by ChIP-seq Signal from ENCODE/Broad
Alu element
Peak triplex
Basic Gene Annotation Set from GENCODE Version 40lift37 (Ensembl 106)
BNIP2
BNIP2
BNIP2
BNIP2
ENSG00000259238

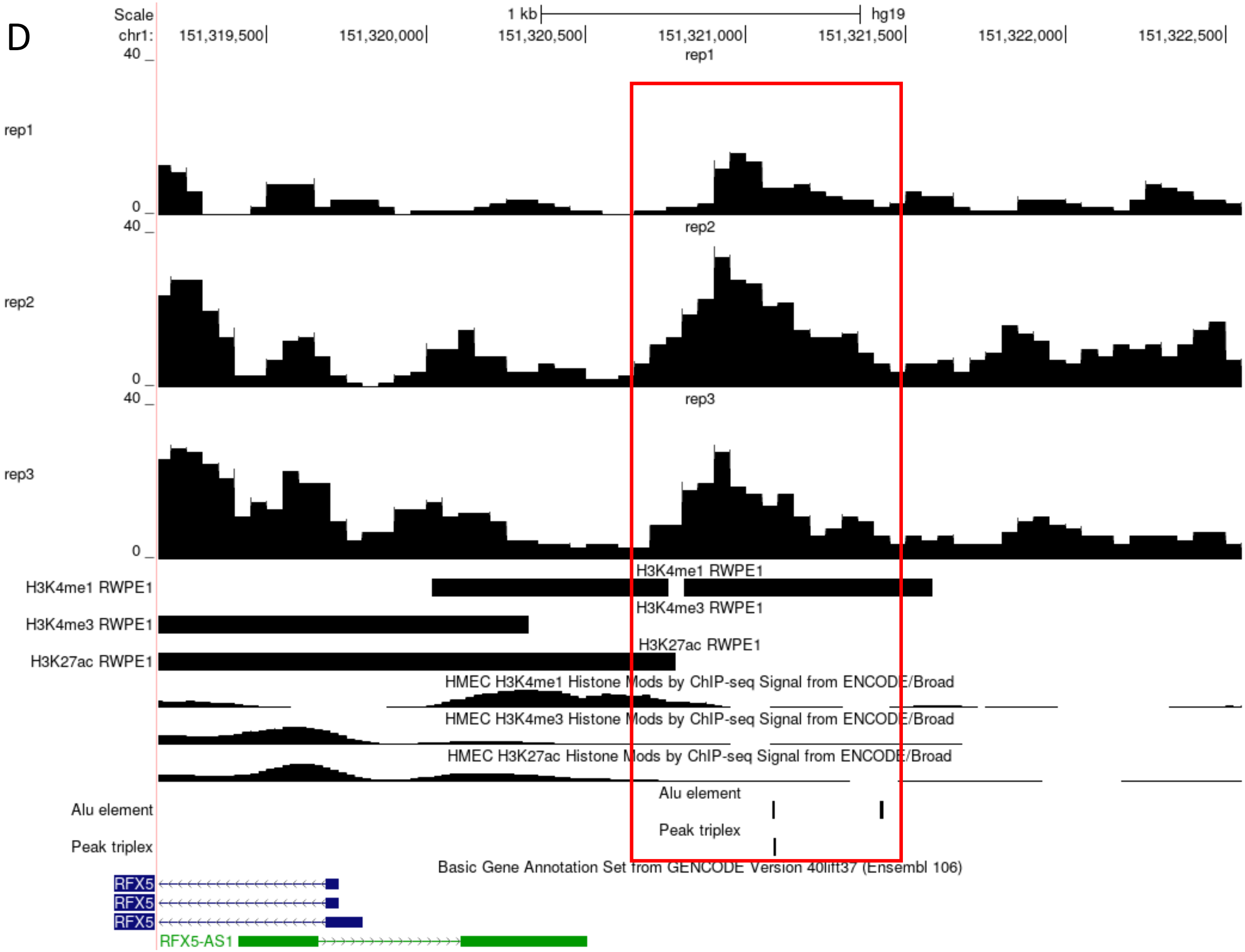

Supplementary Figure S4. Triplex-forming regions (DBS) in the proximal regions of genes bound by *MIR205HG/LEADER* are enriched for *Alu* elements and H3K4me1.
UCSC genome browser tracks of representative genes bound by *MIR205HG* with indication of the triplex-forming region within ChIRP peak and the *Alu* element. The coverage of *MIR205HG* ChIRP peaks from single replicates (rep1, rep2, and rep3) is reported on top, along with the RWPE-1 histone modification (H3K4me1, H3K4me3, and H3K27ac) tracks used for LOLA analysis, which were derived from ChIP-seq data of GSE63094. As further reference, the same histone modification tracks are reported also for human mammary epithelial cells (HMEC), as from ENCODE/Broad. Such cells may be considered as the breast counterpart of RWPE-1 cells.
